# A resolvable controversy in avian conservation

**DOI:** 10.1016/j.heliyon.2024.e35143

**Published:** 2024-07-26

**Authors:** Michael D. Collins

**Affiliations:** Naval Research Laboratory, Washington, DC 20375, USA

## Abstract

Twenty years of squandering an opportunity to save an iconic species from extinction are summarized. In 2005, an article that was featured on the cover of *Science* announced the rediscovery of the Ivory-billed Woodpecker (*Campephilus principalis*) in Arkansas. Despite a subsequent report of sightings in Florida by another group of ornithologists, the persistence of this elusive species became controversial when nobody managed to obtain a clear photo. Video footage that was obtained in Louisiana and Florida between 2006 and 2008 should have resolved the issue, but there was a breakdown in rational discourse after critics became entrenched in the position that the species is extinct. After openly and aggressively attacking relatively weak evidence that was presented in the original article, critics used specious arguments behind the scenes to delay the publication of the strongest evidence for a decade. The bulk of this material was finally published in *Heliyon* in 2017, but the critics have never addressed it. In 2021, the U.S. Fish & Wildlife Service announced a decision to declare the Ivory-billed Woodpecker extinct without addressing the strongest evidence for persistence.

## 1 Introduction

During the past hundred years, the Ivory-billed Woodpecker (*Campephilus principalis*) has persisted in barely detectable numbers and repeatedly been feared extinct only to be rediscovered. Twenty years of squandering an opportunity to save this iconic species from extinction are summarized here. A study that began in Arkansas in 2004 resulted in the first published report of the Ivory-billed Woodpecker by ornithologists in several decades in an article that was featured on the cover of *Science* [1]. Despite a subsequent report of sightings in Florida by another group of ornithologists [2], the persistence of the species became controversial when nobody managed to obtain a clear photo. During three encounters with Ivory-billed Woodpeckers in Louisiana and Florida between 2006 and 2008, I obtained video footage [3], [4], [5], [6], [7], [8], [9], [10] containing the strongest evidence for persistence that has been obtained during the past several decades.

This body of evidence should have resolved the issue, but there was a breakdown in fact-based, logical discourse after critics became entrenched in the position that the Ivory-billed Woodpecker is extinct. Some of the leading science journals had opportunities to foster an open discourse that might have brought the truth to light in the interest of science and conservation, but they instead published negatively biased reports that made it nearly impossible to publish on this topic and thus had the effect of stifling open discourse. After more than forty submissions of the strongest evidence were rejected on the basis of specious arguments, the bulk of this material was finally published in *Heliyon* in 2017 [4]. Despite never addressing the strongest evidence, critics managed to mislead the public and the science community about the status of the Ivory-billed Woodpecker. In 2021, the U.S. Fish & Wildlife Service announced a plan to declare the species extinct without addressing the strongest evidence.

## 2 Observations and evidence

**Figure 1:**
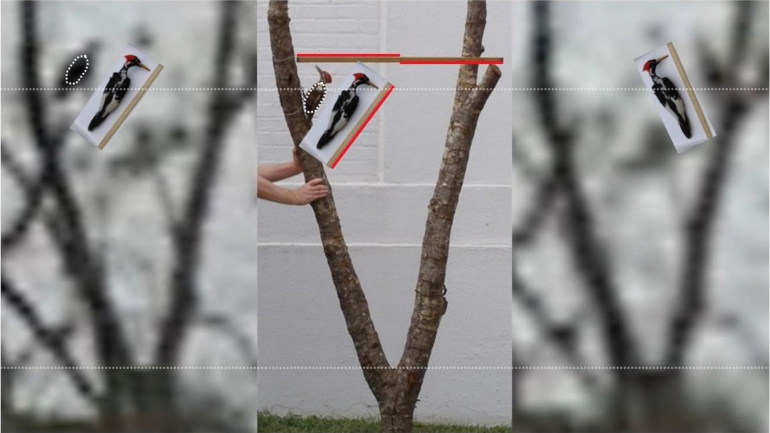
Comparison of the woodpecker in the 2006 video with specimens of the large woodpeckers. Two forks in the tree specimen were used for scaling. The Pileated Woodpecker specimen and a meter stick were mounted on the tree specimen. The Ivory-billed Woodpecker specimen was photographed beside a half meter stick. A dashed curve was used to outline the body of the Pileated Woodpecker specimen and then cut-and-pasted over the body of the woodpecker appearing in the video. The woodpecker in the video dwarfs the Pileated Woodpecker specimen and is comparable in size to an Ivory-billed Woodpecker specimen that is near the maximum size for that species.

At the end of 2005, *Science* selected the Ivory-billed Woodpecker as one of the “Areas to Watch in 2006” [11]. Two months later, I had a flurry of activity during a five-day period in the Pearl River swamp in Louisiana. I had five sightings with excellent views of definitive field marks and flight characteristics. I heard the ‘kent’ calls of the Ivory-billed Woodpecker on two occasions, once coming from two directions at the same time. During one of the encounters, I obtained video footage of a large woodpecker that was perched on a tree, part of which was collected. The tree specimen has widely-spaced forks that facilitated scaling relative to specimens of the Pileated Woodpecker (*Dryocopus pileatus*) and the Ivory-billed Woodpecker. There is no overlap in the body masses of these species, which are the only large woodpeckers north of the Rio Grande. Appearing in Fig. 1 is an update of the comparison in Fig. 2 of Ref. 4. The woodpecker in the video dwarfs a Pileated Woodpecker specimen and is comparable in size to the largest Ivory-billed Woodpecker specimen in the Smithsonian collection, which is near the maximum size for that species. The body of the woodpecker in the video is too large to fit into a Pileated Woodpecker cavity with the largest entrance for that species [10]. The video was analyzed by an avian artist, Julie Zickefoose, whose depiction of the Ivory-billed Woodpecker had recently been featured on the cover of a leading ornithology journal [12]. According to Zickefoose, the large woodpecker appearing in the video has several behaviors and characteristics consistent with the Ivory-billed Woodpecker but not the Pileated Woodpecker [3]. Despite the fact that *Science* had recently expressed a high level of interest in this issue, which was becoming controversial, the Editor was dismissive when informed of new observations and evidence.

**Figure 2:**
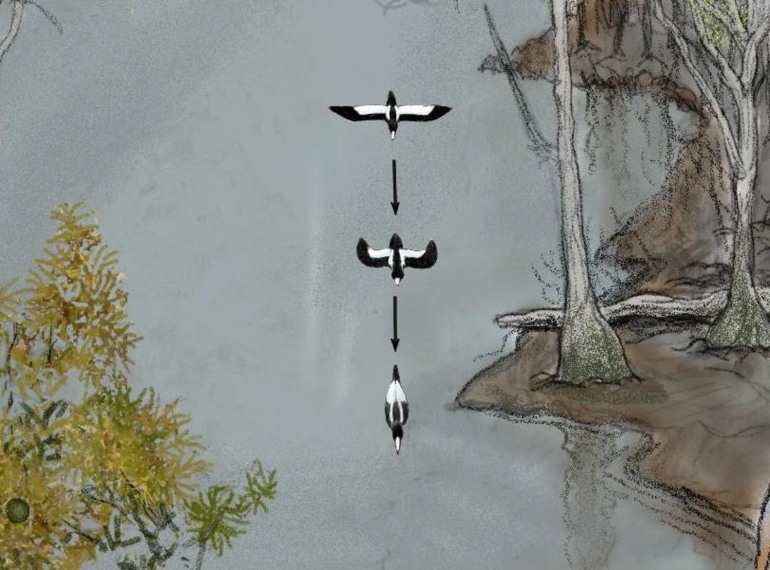
Illustration of the view of an Ivory-billed Woodpecker that flew up the bayou and passed nearly directly below a tree that was used as an observation platform. It was an ideal vantage point for observing the two white stripes on the back and the white trailing edges on the dorsal surfaces of the wings (the Pileated Woodpecker does not have these field marks). This illustration also shows the distinctive wing motion of a large woodpecker in which the wings are folded closed in the middle of each upstroke.

**Figure 3:**
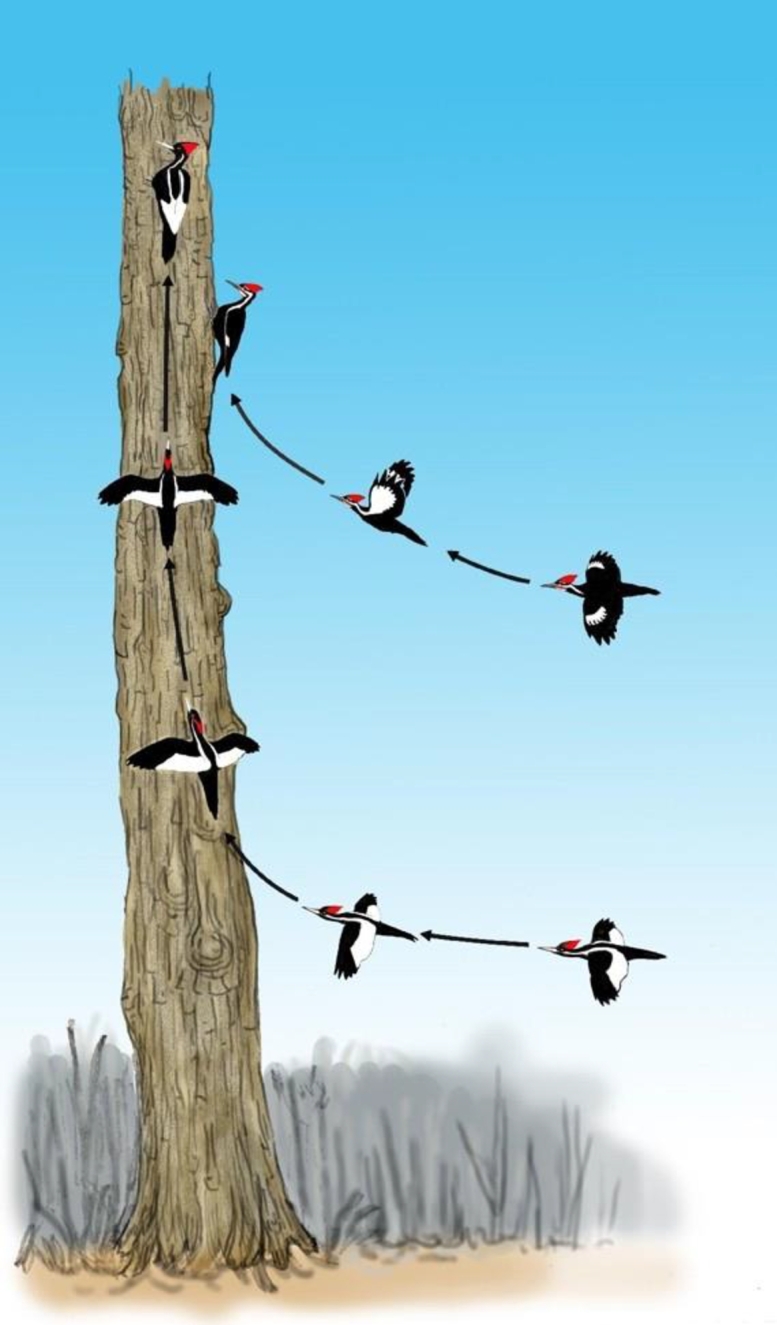
Illustration of upward swooping landings by the large woodpeckers. The Pileated Woodpecker typically swoops upward a short distance and lands on a surface that faces the direction of approach. In two of the upward swooping landings in the 2007 video, the bird appears to rotate about its axis and land on a surface that does not face the direction of approach. A long vertical ascent allows time for such maneuvering. During one of the upward swooping landings, a white trailing edge on the dorsal surface of the right wing was observed through binoculars.

A short distance up the same bayou in March 2008, I obtained a video of a large bird that flew beneath my observation position 75 feet up in a cypress. Since the bird and its reflection from the still surface of the bayou appear in the video, it was possible to pin down locations along the flight path and use this information to measure the flight speed [3] and determine that the wingspan is well over 24 inches [9]. The video shows several wingbeats during which the wings are folded closed in the middle of each upstroke. The two large woodpeckers are the only birds of the region with that wing motion and a wingspan over 24 inches. After digitizing the motion of the wingtips from the video and performing an analysis that he had previously developed and applied to other woodpeckers [13], an expert on woodpecker flight mechanics, Bret Tobalske, also concluded that the bird in the video is a large woodpecker [3]. The wingbeat frequency is about ten standard deviations greater than the mean for the Pileated Woodpecker, which eliminates that species from consideration. The flight speed, narrow wings, and white trailing edges on the dorsal surfaces of the wings are consistent with the Ivory-billed Woodpecker but not the Pileated Woodpecker. The brightness, contrast, and color were adjusted to enhance additional characteristics (a black body and black leading edges on the wings) that are consistent with the Ivory-billed Woodpecker [7]. The video documents that I tracked the flight of the bird for about ten seconds from a favorable vantage point for observing the definitive dorsal field marks (two white stripes on the back and white trailing edges on the wings) as illustrated in Fig. 2. The field marks of the two large woodpeckers are contrasted in Fig. 3.

Geoff Hill is the author of a book on the Ivory-billed Woodpecker [14] and one of the few living ornithologists to have observed that species. After the news came from Arkansas, he followed up on reports from Florida, had a sighting, and reported on a series of sightings and data obtained with his colleagues [2]. After seeing the video from Louisiana that the Editor of *Science* had dismissed in 2006, Hill acknowledged that it was the best evidence that had been obtained up to that point, and he invited me to visit his study area. While working with his group in January 2007, I had an encounter with a distant pair of Ivory-billed Woodpeckers that lasted for more than twenty minutes. I saw definitive field marks of one of the birds through binoculars and watched in awe a series of spectacular swooping flights that were consistent with accounts by Audubon of a flight that is “graceful in the extreme” [15] and by Eckleberry of a landing with a “magnificent upward swoop” [16]. With a high-definition video camera, I captured several of the swooping flights, a double knock that is visible and audible, and takeoffs with deep and rapid wingbeats and loud ‘wooden’ wing sounds [9] that are consistent with an account by Tanner [17]. In Fig. 3, the remarkable upward swooping landings that were observed and captured in the video are contrasted with typical upward swooping landings by the Pileated Woodpecker. The audio track contains several pairs of spring-like sounds that are of unknown origin, but one of them coincides with a sudden change in direction at the beginning an upward swoop [9]. It takes more of an effort to understand the 2007 video relative to the others, but it contains perhaps the most interesting footage of the Ivory-billed Woodpecker that has ever been obtained (no flights appear in the only existing historical film, which was obtained in 1935).

In addition to evidence, the videos provide insights into the flights and other behaviors of this fascinating species. The videos show several types of flight, including a cruising flight, a short flight between limbs, takeoffs with deep and rapid wingbeats, upward swooping landings, downward swooping takeoffs, and a takeoff with rapid wingbeats that immediately transitions into an upward swooping flight. The 2007 video shows the only double knock of an Ivory-billed Woodpecker that has ever been filmed, which led to the discovery (based on the concept of a harmonic oscillator) that the second impact is a rebound rather than the result of two deliberate blows [5]; after this hypothesis was inspired by the 2007 video, it was confirmed for other *Campephilus* woodpeckers that communicate with double knocks. Several types of calls were observed during the study in the 1930s, but only the kent calls were captured in the historical recording. During two encounters in 2006, high-pitched calls were heard coming from the direction of an alarmed Ivory-billed Woodpecker, and several of them were captured in the 2006 video [3]. These calls match the description of a high-pitched alarm call, and they have a spectrum consisting of simultaneously excited harmonics that is consistent with the spectrum of kent calls.

The videos provide a powerful body of evidence, but the dozens of sightings during the searches in Arkansas, Florida, and Louisiana should also be considered. The Ivory-billed Woodpecker is a large bird with distinctive and prominent field marks. It is not plausible to dismiss as a series of mistakes that many sightings of such a bird by observers who were experienced at identifying birds in the field, knowledgeable of the field marks of the Ivory-billed Woodpecker, and acclimated to southern swamp forest habitats and the species that regularly occur in them.

## 3 Folly and shenanigans

In August 2007, *Science* published a report on the status of this issue [18] that provided a platform for unsupported opinions and falsely stated that no new evidence had been obtained. There was no mention of the strongest evidence that has been obtained during the past several decades, which would have been more relevant to the debate than opinions such as the following: “It's just a perfect recipe for your brain to fill in the gaps,” Sibley says. “You get a brief glimpse and an impression,... and your brain turns it into an ivory-billed woodpecker.” This characterization of sightings by an avian artist does not apply to any of my sightings, some of which are supported by video evidence that nobody has been able to refute; a more relevant opinion from an avian artist to include in the report would have been the assessment of the 2006 video by Zickefoose. The report contains a critical comment by Hill, who described a video that was presented in the original paper as “one of the most unfortunate things to ever happen to the [Cornell] Laboratory of Ornithology.” There was nothing wrong with including Hill's comment, but it would have been more enlightening to include his assessment of the videos that were obtained in Louisiana and Florida. After studying those videos, Hill concluded that they are “very convincing.” In February 2010, *Nature* published a report on the status of this issue [19], which mentioned that a non-scientist had faked a photo but made no mention of the strongest evidence. The negatively biased reports that appeared in the leading science journals caused the issue to become deeply marginalized.

In the years that followed, it took more than forty submissions to get the strongest evidence published. Behind the scenes, critics used specious arguments to delay publication for a decade. Specific examples discussed in Refs. [6] and [9] reveal an apparent lack of knowledge of basic facts about topics such as the Ivory-billed Woodpecker and avian flight mechanics. Critics also made unfounded assertions of fraud. A submission to the *Proceedings of the National Academy of Sciences* in 2009 was rejected on the basis of an assertion that the speed of the 2008 video had been altered in order to double the apparent wingbeat frequency and flight speed; this amounted to a concession that the evidence cannot be explained away with logical reasoning. It would have been easy to confirm that the video is legitimate, but the Editor declined to check facts. A suggestion that the tree specimen used in the size comparison for the 2006 video is not from the actual perch tree was debunked using a series of photos of the tree that were taken shortly after the video was obtained [9]. A suggestion that the 2006 video was obtained in the tropics and shows a different *Campephilus* woodpecker was debunked by launching a drone at the site, where trees in the video were still recognizable, and flying it upward until landmarks at the nearby Stennis Space Center came into view [9].

Each of the videos shows field marks, body proportions, flights, and other behaviors that are consistent with the Ivory-billed Woodpecker but no other species. The issue might have been resolved more than fifteen years ago if there had been an open discourse on this evidence. After the eventual publication of the strongest evidence, critics avoided openly addressing it. The pattern of openly attacking a convenient target (the relatively weak evidence that was presented in the original paper) and making it the focus, working behind the scenes to delay the publication of the strongest evidence, and then avoiding any open discourse on the strongest evidence after its publication is suggestive of an agenda that has nothing to do with seeking the truth.

In September 2021, the U.S. Fish & Wildlife Service announced a decision to declare the Ivory-billed Woodpecker extinct [20]. The decision came in the wake of the most massive spike in published reports of sightings and evidence during the past several decades, and it was made without addressing the strongest evidence. In May 2023, there was a report of sightings and evidence from a different location in Louisiana [21], including videos that were purported to show Ivory-billed Woodpeckers on the basis of apparent white markings. The videos were obtained from above on sunny days. Under those conditions, apparent white markings often correspond to solar glare rather than actual field marks. In one of the videos, prominent white patches appear on both of the wings and on the tail, but both of the large woodpeckers have black tails. The bird in one of the videos has field marks and flight characteristics consistent with the Pileated Woodpecker but not the Ivory-billed Woodpecker [10].

## 4 Summary

The Ivory-billed Woodpecker has a long history of elusiveness, with the first rediscovery occurring a hundred years ago [22]. The remarkable elusiveness of this bird is due to a ‘perfect storm’ combination of factors related to behavior and habitat [4], [6]. It is known from historical accounts that these birds are wary and easy to overlook in the forest canopy; they roam over wide areas in vast swamp forests that are difficult to access and where visibility is limited to short distances. During a study in the 1930s, clear photos were obtained at one of the last known nests [17]. It would have been desirable to obtain photos under a variety of conditions, but only a few poor-quality photos were obtained away from the nest during that study.

Nobody was able to obtain a clear photo during intensive, multi-year efforts in Arkansas, Florida, and Louisiana. Nobody is likely to obtain such ideal evidence unless a nest is discovered, but we already have evidence that is sufficient to resolve the question of persistence and justify the establishment of the first-ever conservation program for this critically endangered and long-neglected species. There is no excuse for pushing the narrative that the species is extinct without addressing the strongest evidence for persistence, which is not difficult to understand.

The 2006 video shows a large woodpecker that, according to an avian artist who specializes in the Ivory-billed Woodpecker, has several characteristics and behaviors consistent with that species but not the Pileated Woodpecker. The bird in that video was perched on a tree, part of which was collected. Two widely-spaced forks in the tree specimen provide a reliable length scale, and the woodpecker in the video is comparable in size to the largest Ivory-billed Woodpecker specimen in the Smithsonian collection and appears to have a larger body than any Pileated Woodpecker.

The 2008 video shows a bird in flight with a wingspan well over 24 inches and a flap style in which the wings are folded closed in the middle of each upstroke. The two large woodpeckers are the only species of the region with that combination of size and wing motion, and an expert on woodpecker flight mechanics concluded that the bird in that video is a large woodpecker on the basis of an analysis of the motion of the wingtips through a flap cycle. After reaching the conclusion that the bird in the video is a large woodpecker, the final conclusion follows from the fact that the wingbeat frequency is about ten standard deviations greater than the mean wingbeat frequency of the Pileated Woodpecker. In addition, the field marks, high flight speed, and narrow wings are consistent with the Ivory-billed Woodpecker but not the Pileated Woodpecker.

In order to understand the 2007 video, it is necessary to have some familiarity with the flights and other behaviors of the Ivory-billed Woodpecker and other species of the region. Since that video shows a series of events involving field marks, body proportions, flights, and other behaviors consistent with the Ivory-billed Woodpecker but no other species of the region, it is easy to understand the power of this evidence in terms of a basic concept: the probability of a series of unlikely events quickly becomes astronomically small as the number of events increases.

## CRediT authorship contribution statement

**Michael D. Collins:** Writing – review & editing, Writing – original draft, Visualization, Validation, Supervision, Software, Resources, Project administration, Methodology, Investigation, Funding acquisition, Formal analysis, Data curation.

## Declaration of Competing Interest

The authors declare that they have no known competing financial interests or personal relationships that could have appeared to influence the work reported in this paper.

## Data Availability

The data associated with this study have been deposited in a publicly available repository. It may be accessed at https://doi.org/10.5061/dryad.8w9ghx3hp.
